# Environmental costs and benefits of growing *Miscanthus* for bioenergy in the UK

**DOI:** 10.1111/gcbb.12294

**Published:** 2015-08-18

**Authors:** Jon P. McCalmont, Astley Hastings, Niall P. McNamara, Goetz M. Richter, Paul Robson, Iain S. Donnison, John Clifton‐Brown

**Affiliations:** ^1^ Institute of Biological, Environmental and Rural Sciences (IBERS)Aberystwyth UniversityGogerddan, AberystwythWalesSY23 3EQUK; ^2^ Institute of Biological and Environmental ScienceUniversity of Aberdeen24 St Machar DriveAberdeenAB24 3UUUK; ^3^ Centre for Ecology & HydrologyLancaster Environment CentreLibrary Avenue, BailriggLancasterLA1 4APUK; ^4^ Rothamsted ResearchWest CommonHarpenden, HertfordshireAL5 2JQUK

**Keywords:** biodiversity, bioenergy, crop modelling, energy crops, GHG, land‐use change, *Miscanthus*, perennial grasses, plant ecophysiology, renewable energy

## Abstract

Planting the perennial biomass crop *Miscanthus* in the UK could offset 2–13 Mt oil eq. yr^−1^, contributing up to 10% of current energy use. Policymakers need assurance that upscaling *Miscanthus* production can be performed sustainably without negatively impacting essential food production or the wider environment. This study reviews a large body of *Miscanthus* relevant literature into concise summary statements. Perennial *Miscanthus* has energy output/input ratios 10 times higher (47.3 ± 2.2) than annual crops used for energy (4.7 ± 0.2 to 5.5 ± 0.2), and the total carbon cost of energy production (1.12 g CO_2_‐C eq. MJ^−1^) is 20–30 times lower than fossil fuels. Planting on former arable land generally increases soil organic carbon (SOC) with *Miscanthus* sequestering 0.7–2.2 Mg C4‐C ha^−1^ yr^−1^. Cultivation on grassland can cause a disturbance loss of SOC which is likely to be recovered during the lifetime of the crop and is potentially mitigated by fossil fuel offset. N_2_O emissions can be five times lower under unfertilized *Miscanthus* than annual crops and up to 100 times lower than intensive pasture. Nitrogen fertilizer is generally unnecessary except in low fertility soils. Herbicide is essential during the establishment years after which natural weed suppression by shading is sufficient. Pesticides are unnecessary. Water‐use efficiency is high (e.g. 5.5–9.2 g aerial DM (kg H_2_O)^−1^, but high biomass productivity means increased water demand compared to cereal crops. The perennial nature and belowground biomass improves soil structure, increases water‐holding capacity (up by 100–150 mm), and reduces run‐off and erosion. Overwinter ripening increases landscape structural resources for wildlife. Reduced management intensity promotes earthworm diversity and abundance although poor litter palatability may reduce individual biomass. Chemical leaching into field boundaries is lower than comparable agriculture, improving soil and water habitat quality.

## Introduction

The IPCC 5th report (IPCC, 2014) makes clear that it is *extremely likely* that cumulative anthropogenic greenhouse gas emissions have led to unequivocal climate warming with temperature and precipitation extremes seen since the 1950s that are unprecedented over millennia. The report states with *high confidence* that without additional mitigation efforts, climate warming will more likely than not exceed 4 °C above pre‐industrial levels by 2100; extremes of weather resulting from this would lead to ‘substantial species extinctions, global and regional food insecurity, and consequential constraints on human activities’ with the highest relative price to be paid by those least responsible for the problem. The IPCC states categorically that limiting these impacts to more manageable levels requires net global CO_2_ emission to decrease to zero in the next few decades through rapid decarbonization of energy production. Sustainable biomass offers the almost unique opportunity to provide storable, flexible use of fuel that can be readily converted to heat, electricity, or even liquid transport fuels and is the single option that might provide a future mechanism to remove atmospheric carbon by capture and storage (CCS) (ETI 2015). Over the past 20 years, fairly comprehensive field data have become available for the clone‐based interspecies hybrid *M. giganteus*. This review examines the environmental benefits and trade‐offs associated with large‐scale planting of *Miscanthus* for UK bioenergy, now made possible by recent breeding of seed‐based hybrids in the UK and USA. The review focusses on environmental impacts of field production; it does not cover wider economic analyses that are also important determinants of commercial uptake.

## Renewable fuels

**Figure 1:**
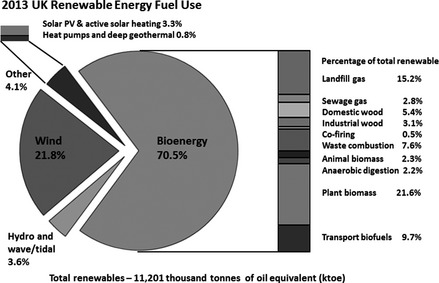
Current UK renewable energy use as of end 2013, up 30% between 2012 and 2013, supplying 14.9% of UK electricity. Bioenergy contributes 70.5% of total renewable with plant biomass alone contributing 21.6% at 3.9 Mt which was primarily imported.

Use of renewable energy in the UK mix was up 30% from 2012 to 2013 (see Fig. 1); it supplied 14.9% of UK electricity which was 5.2% of total energy (DUKES 2014a), well short of the UK 2012 Bioenergy Strategy target of 15% of total energy by 2020 (DECC, 2012). Plant biomass supplied 21.6% of total renewables which offset 2079 kt of oil equivalent electricity and 339 kt oil eq. heat (DUKES 2014b). Despite the replacement of 2.4 million tonnes of oil use, domestic biomass production remains at a low level compared to total energy demand. Only 0.05 Mha, or 0.8%, of UK arable land was used for bioenergy production in 2013 with 0.03 Mha being used to produce maize for anaerobic digestion (DEFRA 2014a). Results from the OFGEM biomass sustainability data set (Ofgem 2014), excluding liquid feedstocks or anaerobic digestion, showed the UK burnt 3.9 Mt of plant biomass for electricity in 2013; of this, 1.8 Mt was produced in the UK with the majority being wood products. Home grown dedicated energy crops provided only 56 kt: 47 kt of *Miscanthus* and 9 kt of SRC willow.

## Biomass crops

While the initial premise regarding bioenergy was that carbon recently captured from the atmosphere into plants would deliver an immediate reduction in GHG emission from fossil fuel use, the reality proved less straightforward. Studies suggested that GHG emission from energy crop production and land‐use change might outweigh any CO_2_ mitigation (Searchinger *et al*., 2008; Lange, 2011). Nitrous oxide (N_2_O) production, with its powerful global warming potential (GWP), could be a significant factor in offsetting CO_2_ gains (Crutzen *et al*., 2008) as well as possible acidification and eutrophication of the surrounding environment (Kim & Dale, 2005). However, not all biomass feedstocks are equal, and most studies critical of bioenergy production are concerned with biofuels produced from annual food crops at high fertilizer cost, sometimes using land cleared from natural ecosystems or in direct competition with food production (Naik *et al*., 2010). Dedicated perennial energy crops, produced on existing, lower grade, agricultural land, offer a sustainable alternative with significant savings in greenhouse gas emissions and soil carbon sequestration when produced with appropriate management (Crutzen *et al*., 2008; Hastings *et al*., 2008, 2012; Cherubini *et al*., 2009; Dondini *et al*., 2009a; Don *et al*., 2012; Zatta *et al*., 2014; Richter *et al*., 2015). Greenhouse gas mitigation is a primary concern in bioenergy production but is not the only consideration, particularly for large‐scale land‐use transitions. Bioenergy supply chains must be energetically favourable, maintain or increase soil carbon, and be cost effective and environmentally sustainable without interfering with essential food production (Tilman *et al*., 2009; Valentine *et al*., 2012). *Miscanthus × giganteus* (hereafter *Miscanthus*), a low‐input, fast‐growing perennial energy grass, is seen to offer an attractive alternative biomass crop (Lewandowski *et al*., 2003; Harvey, 2007; Heaton *et al*., 2008, 2010; Zhuang *et al*., 2013) with energy output/input ratios around ten times that of annual energy crops (Felten *et al*., 2013) and significant potential to reduce fossil fuel CO_2_ emission (Clifton‐Brown *et al*., 2004, 2007; Hillier *et al*., 2009). Felten *et al*. (2013) compared energy balances for oil seed rape (OSR), maize, and *Miscanthus* and found output/input ratios of 4.7 ± 0.2, 5.5 ± 0.2, and 47.3 ± 2.2, respectively.

## Potential UK land availability

**Figure 2:**
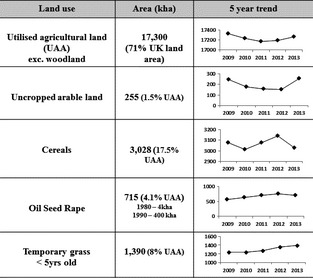
2013 extent and 5‐year trends in major UK agricultural land areas, 0.26 Mha of arable were uncropped in 2013 due to poor weather in 2012 preventing annual cultivation.

The 2007 UK biomass strategy (DEFRA, 2007) set a target of 0.35 Mha of UK agricultural land growing perennial energy crops by 2020; this would be part of an overall one million hectares in biofuel and energy crop production. The 2012 UK Bioenergy Strategy (DECC, 2012) suggests that the potential land available specifically for *Miscanthus* that would not impinge on food production is in the range of 0.72–2.8 Mha which is well above the 2007 target (Fig. 2 puts the 0.35 Mha into context by showing current UK agricultural land use and 5 year trends). These strategy reports stress that while some energy crops may reduce soil erosion, improve biodiversity, and aid fuel security, production must take place ‘…in those parts of the UK where it makes sense…’

**Figure 3:**
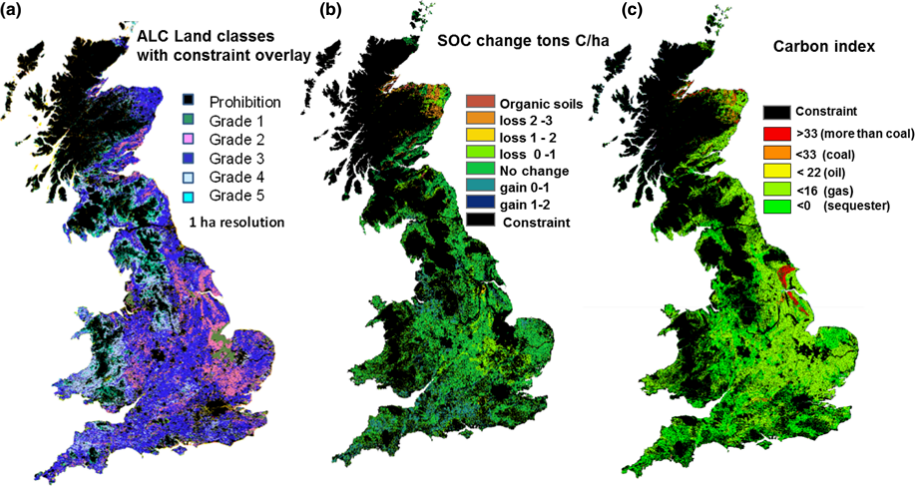
(a) shows distribution of agricultural land classes (ALC) with excluded areas in black following Lovett *et al*. (2014). (b) shows the map of modelled annual change in soil carbon following land‐use change from existing agriculture to *Miscanthus* outside these areas from Milner *et al*. (2015), and (c) shows the potential carbon intensity index, in g CO_2_‐C equivalent per MJ energy in the furnace, compared to coal (33), oil (22), and North Sea gas (16). The *Miscanthus* carbon intensity is calculated considering rhizome propagation, 2‐year establishment, pelletized fuel, and 100 km of transportation.

Lovett *et al*. (2009) reported that 0.35 Mha of *Miscanthus* could be easily accommodated in the UK. Initially considering just England, using a GIS approach, they produced a constraint map based on 11 preclusion factors covering biophysical, social, and environmental considerations, for example high soil carbon content, cultural or natural heritage, and urban centres, with a final constraint that only poorer quality land in agricultural land class (ALC) grades 3 or 4 would be considered, excluding higher grades 1 and 2 and the worst grade 5. Results suggested potential land availability for *Miscanthus*, of 3.12 Mha, around one‐quarter of total English land area. The authors point out that the 0.35 Mha target represents only 11.6% of this and planting *Miscanthus* on this more marginal agricultural land would not impinge on essential food production. Lovett *et al*. (2014) expands the GIS constraint mapping to include Scotland and Wales (see Fig. 3a). Here, results suggested 8.5 Mha potentially suitable for growing *Miscanthus* or SRC willow/poplar, applying the extra restriction to ALC grade 3 or worse reduced this to 6.4 Mha. Grade 3 agricultural lands represent the majority of UK farmland and covered around 59% of the total 8.5 Mha identified in this study, excluding this and restricting planting to the very worst agricultural land grades 4 and 5 left 1.4 Mha, four times the 0.35 Mha target.

### Land‐use change

Land‐use change is central to UK agriculture, field crop species and farming practices are in constant flux, and land usage will typically follow an economic rationale within the constraints of the EU's Common Agricultural Policy. In 1993, to curb EU overproduction of food, farmers set aside a minimum of 15% of their cropped land; by 2000, this had dropped to 10% and was zero by 2008. In England alone, from 2000 to 2006, the average land area set aside was 0.57 Mha (DEFRA 2014b). The days of set aside are over, agricultural production must increase worldwide to meet growing demands for both food and energy; accommodating this while avoiding increased exploitation of natural lands requires a move towards more sustainable intensification (Tilman *et al*., 2011; Godfray & Garnett, 2014). Concentrating agronomic effort and resources away from the least productive 10% of farmed area to more productive land while retaining a low‐input, high‐output perennial energy crop on these poorer areas could offer a mechanism for both intensification and diversification within farms. Farmers might identify areas of their farms where yields of more conventional crops are at their lowest, and bought at the expense of high effort and chemical input while detracting effort from their more productive land, and give this area over to at least one economic cropping cycle of a low‐input perennial. *Miscanthus* can improve overworked or difficult soils by acting as a long‐term break crop, increasing soil carbon, organic matter, and earthworm diversity (Kahle *et al*., 2001; Hansen *et al*., 2004; Felten & Emmerling, 2011). Perhaps an ideal situation would see the cycling of this long‐term break crop around the farm area with conventional crop rotations following to take advantage of the improved organic matter and soil structure. Figure 2 shows a summary of some of the main UK agricultural land uses on areas that might be suitable for *Miscanthus* production and the variability of their extent between 2009 and 2013 (DEFRA 2014c). Of the 17.3 Mha of utilized agricultural area (UAA) in the UK, around 3 Mha (17.5%) were in cereal production in 2013, and this area varied between 3 and 3.2 Mha over the last 5 years with a not unusual 0.26 Mha being completely uncropped in 2013 due to high rainfall, preventing planting in autumn 2012. The area of temporary grassland less than 5 years old, perhaps a prime candidate for *Miscanthus* production in western areas of the UK, has been steadily increasing over the last 5 years, currently 1.39 Mha (8% of UAA), up from 1.24 Mha in 2009. The current extent of oil seed rape (OSR) production, at 0.72 Mha, is more than double the *Miscanthus* target. Land cover for this conspicuous, high‐input crop had risen from being almost unknown in 1980 to 0.40 Mha by 1990 and covering more than 0.70 Mha by 2013 (DEFRA 2014d). Modelling studies (Lovett *et al*., 2009; Hastings *et al*., 2013) show that the mean yield of *Miscanthus* on grade 3b, 4, and 5 land outside the excluded areas is around 10 tons DM ha^−1 ^yr^−1^, and the 2007 Biomass Strategy target of 0.35 Mha could produce up to 70 PJ energy, equivalent to 1.67 Mt of oil or 1.17% of total UK energy. The 2012 Bioenergy Target range of 0.72–2.8 Mha would produce 3.44–13.38 Mt oil eq. (2.41–9.39% of total energy). Agricultural land is a finite resource in the UK, providing a range of ecosystem services: from food and energy to culture and leisure with impacts on water quality and natural habitats. Milner *et al*. (2015) offer a comprehensive ‘threat matrix’ quantifying the potential impact on a range of ecosystem services across the UK from land‐use change to perennial biomass crops. They concluded that there was little difference between *Miscanthus* and SRC when planted on lower grade land with both offering positive improvements in service provision although climate‐driven yield estimates and previous land‐use were key factors. Optimization of land utilization at a national scale is essential although currently lacking, if land is to be used for producing energy, then policy should aim to produce the maximum amount of energy per ha within the context of wider ecosystem service provision. In terms of energy production intensity, *Miscanthus* biomass produces more net energy per hectare than other bioenergy crops at around 200 GJ ha^−1 ^yr^−1^, especially arable [maize for biogas 98, oil seed rape for biodiesel 25, wheat and sugar beet ethanol 7–15 (Hastings *et al*., 2012)]. Felten *et al*. (2013) calculated similar figures, reporting 254 GJ ha^−1^ yr^−1^ for *Miscanthus*. Energy production intensity calculated for woody perennials can vary significantly by area (Bauen *et al*., 2010) with yield predictions largely driven by future climate projections (Hastings *et al*., 2013). Tallis *et al*. (2013) showed that in the right circumstances, even old varieties of SRC willow can exceed 150 GJ ha^−1^ yr^−1^, suggesting that planting combinations of crops may be most efficient for overall energy production.

## Soil carbon

More than twice as much carbon is stored in the world's soils compared to vegetation or atmosphere (Post *et al*., 1990; Lal, 2004; Cox *et al*., 2011; Scharlemann *et al*., 2014). It is critically important to understand the impact of large‐scale agricultural land‐use change on these storage reservoirs. Although extending the capacity of European soils to sequester carbon may be limited relative to overall CO_2_ emissions (Smith *et al*., 1997; Mackey *et al*., 2013), the potential for losing soil carbon through misplaced land‐use change could be far more significant. Any soil disturbance, such as ploughing and cultivation, is likely to result in short‐term respiration losses of soil organic carbon, decomposed by stimulated soil microbe populations (Cheng, 2009; Kuzyakov, 2010). Annual disturbance under arable cropping repeats this year after year resulting in reduced SOC levels. Perennial agricultural systems, such as grassland, have time to replace their infrequent disturbance losses which can result in higher steady‐state soil carbon contents (Gelfand *et al*., 2011; Zenone *et al*., 2013). Upscale predictions of these carbon deltas rely on measurements of sample data informing and validating process models (Dondini *et al*., 2009a; Zatta *et al*., 2014; Agostini *et al*., 2015). However, collecting enough samples to determine the significance level of any observed change can be challenging (Kravchenko & Robertson, 2011) and it is extremely rare to find baseline soil samples taken prior to land‐use change. Adjacent reference sites of the previous land‐use are generally taken to represent baseline soil conditions although these may not necessarily represent initial conditions accurately. Richter *et al*. (2015) had the rare opportunity to compare *Miscanthus* soils after 14 years to both archived baseline and an adjacent reference site, though only at 0–30 cm; they found that using the reference site would have suggested greater declines in original SOC than were seen when compared to the actual baseline. With these limitations in mind, we report here results from empirical sample data and discuss whether some clear trends emerge.

Table 1 summarizes nine soil sampling studies of land‐use transition from both arable and grassland with SOC stocks compared to adjacent land. Across these nine studies, 21 comparisons were made between *Miscanthus* plantations and grassland (7) or arable (14) with plantation ages ranging from 3 to 19 years. Direct comparisons of absolute numbers between studies are problematic. Some studies (Hansen *et al*., 2004; Clifton‐Brown *et al*., 2007; Schneckenberger & Kuzyakov, 2007; Dondini *et al*., 2009b; Poeplau & Don, 2014) sampled only single sites within each comparison or age class, while others (Felten & Emmerling, 2012; Zimmermann *et al*., 2012) had multiple sites within each comparison. Only two studies (Zatta *et al*., 2014; Richter *et al*., 2015) had access to a baseline soil archive collected prior to the land‐use change; both these studies investigated the impact on soil carbon of different *Miscanthus* genotypes, while others were limited to *M x giganteus*. Sample depths also varied widely as did management regimes with some sites fertilized and others not. Despite this variability, it seems likely that arable land converted to *Miscanthus* will sequester soil carbon; of the 14 comparisons, 11 showed overall increases in SOC over their total sample depths with suggested accumulation rates ranging from 0.42 to 3.8 Mg C ha^−1^ yr^−1^. Only three arable comparisons showed lower SOC stocks under *Miscanthus,* and these suggested insignificant losses between 0.1 and 0.26 Mg ha^−1 ^yr^−1^.

**Table 1:** Baselines and changes in soil organic carbon (SOC), soil core analysis under land‐use change to Miscanthus from arable and grassland. Results compared to concurrent reference sites taken to represent baseline conditions before conversion in all cases except Zatta *et al*. (2014) who compared results to baseline soil archive. Richter *et al*. (2015) also offer baseline soil archive comparison but not to total sample depth, and results shown here are from their reference site (see text for details). Individual results shown are as reported from individual sites or means from multiple sites in the same transition class/age group; superscripts in location field indicate site numbers in each comparison

Location	Mean annual air temp. (^o^C)/rainfall (mm)	Soil texture (0–30 cm)	Mean harvest yield (Mg DM ha^−1^ yr^−1^)	Total sample depth (cm)	Plantation age (years)	Land‐use prior to Miscanthus plantation	Comparison land use	Total SOC under Miscanthus to sample depth (Mg C ha^−1^)	Total SOC under control to sample depth (Mg C ha^−1^)	Direction of suggested SOC change	Net SOC (C3 and C4) accumulation rate since planting (over total sample depth) (Mg C ha^−1^ yr^−1^)	C4‐C accumulation over sample depth (Mg C ha^−1^ yr^−1^)	References
Ireland^1^	9.9/1004	Loam/sandy loam	13.4	30^FD^	15	Grassland	Grassland	64	59.7	up	0.29^ns^	0.6	Clifton‐Brown *et al*. (2007)
Ireland^8^	9.3/822	Sandy loam	na	30^FD^	3	Grassland	Grassland	79.6	80.625	down	−0.34^ns^	0.9	Zimmermann *et al*. (2012)
UK^1^	9.9/1216	Sandy clay loam	14.5	30^ESM^	6	Grassland	Grassland	71.6	78.8	down	−1.2^ns^	1.25	Zatta *et al*. (2014)
Germany^1^	8.7/679	Loam	na	100^FD^	9	Grassland	Grassland	112	121	down	−1^ns^	Mg ha^−1^ na	Schneckenberger & Kuzyakov (2007)
Germany^1^	8.7/548	Sandy loam	na	100^FD^	12	Arable/fallow	Grassland	70	64	up	0.5^ns^	Mg ha^−1^ na	Schneckenberger & Kuzyakov (2007)
Denmark^1^	7.4/705	Coarse loamy sand	12.6	100^FD^	9	Arable	Grassland	91	91	no change	0^ns^	0.78	Hansen *et al*. (2004)
Denmark^1^	7.4/706	Coarse loamy sand	14.1	100^FD^	16	Arable	Grassland	106	91	up	0.94*	1.13	Hansen *et al*. (2004)
Ireland^1^	9.3/830	Sandy loam	16	60^FD^	14	Arable	Arable	131.3	105.8	up	1.82*	3.2	Dondini *et al*. (2009a,b)
Germany^4^	10.5/761	Loamy sand	15	150^FD^	2	Arable	Arable	92.9	90.5	up	1.2^ns^	1.4	Felten & Emmerling (2012)
Germany^2^	10.5/762	Loamy sand	15	150^FD^	5	Arable	Arable	109.5	90.5	up	3.8^ns^	0.68	Felten & Emmerling (2012)
Germany^7^	10.5/763	Loamy sand	15	150^FD^	16	Arable	Arable	112.5	90.5	up	1.375*	1.03	Felten & Emmerling (2012)
Denmark^1^	7.4/707	Coarse loamy sand	12.6	100^FD^	9	Arable	Arable	91	92	down	−0.1^ns^	0.78	Hansen *et al*. (2004)
Denmark^1^	7.4/708	Coarse loamy sand	14.1	100^FD^	16	Arable	Arable	106	92	up	0.88*	1.13	Hansen *et al*. (2004)
Netherlands^1^	8/760	na	13	80^FD^	11	Arable	Arable	93.9	77.6	up	1.48^ns^	0.70	Poeplau & Don (2014)
Germany^1^	7.3/550	na	15	80^FD^	15	Arable	Arable	147.8	150.3	down	−0.17^ns^	0.37	Poeplau & Don (2014)
Denmark^1^	7.9/859	na	14	80^FD^	18	Arable	Arable	96.5	89	up	0.42^ns^	0.54	Poeplau & Don (2014)
Switzerland^1^	8.4/1185	na	14	80^FD^	17	Arable	Arable	133	116.2	up	0.99*	0.80	Poeplau & Don (2014)
Germany^1^	9/707	na	15	80^FD^	19	Arable	Arable	94.8	77.6	up	0.91*	0.93	Poeplau & Don (2014)
Switzerland^1^	9/860	na	14	80^FD^	15	Arable	Arable	108.7	85.2	up	1.57*	0.88	Poeplau & Don (2014)
Ireland^8^	9.3/823	Sandy loam	na	30^FD^	3	Arable	Arable	64.52	60.51	up	1.34^ns^	0.62	Zimmermann *et al*. (2012)
UK^1^	na	Silty clay loam	8.75	100^ESM^	14	Arable	Arable	106.9	110.6	down	−0.26^na^	1.10	Richter *et al*. (2015)

FD, fixed depth sampling; ESM, equivalent soil mass; ns, not significant; na, unavailable.

Zatta and Richter are mean results from several genotypes.

Superscript number in location field indicates number of sites included in the result.

Superscript in SOC accumulations indicates significance of change where available, * = *P* < 0.05.

The grassland to *Miscanthus* comparisons showed three increases, three decreases, and one no change in soil C stocks. no doubt complicated by the *Miscanthus* being planted on former arable, arable/fallow, or grassland despite being compared to long‐term grassland, whereas all comparisons to arable were planted on former arable land. The range of gains and losses was relatively small, −1 to 0.94 Mg C ha^−1^ yr^−1^ with only the increase of 0.94 Mg ha^−1^ yr^−1^ shown to be significant (Hansen *et al*., 2004). Another study, not included in Table 1 due to incompatibility of units (Kahle *et al*., 2001), primarily compared plant derived organic matter between *Miscanthus* and grassland but also sampled for organic carbon at four sites in Germany over multiple years making a total of 12 comparisons. Of these, 8 showed higher SOC stocks under *Miscanthus* compared to the grassland with 5 of these being shown to be highly significant (*P* < 0.01), only one site showed lower concentrations of SOC across 2 years of sampling. One literature review (Anderson‐Teixeira *et al*., 2009) felt confident to suggest that conversion of temperate grassland to *Miscanthus* would see an eventual increase in SOC, but the results here would suggest that there are enough uncertainties to prevent making such an outright assertion, and it is perhaps safer to suggest that over the lifetime of the crop SOC stocks in the soil profile would be at least maintained. In unpublished work, R. Rowe, A.M. Keith, D. Elias, M. Dondini, P. Smith, J. Oxely and N.P. McNamara (2015, in submission) investigated multiple paired comparisons between *Miscanthus* and grassland (nine sites, mean age 7 years) and *Miscanthus* and arable (11 sites, mean age 6.5 years) and reported the results of soil carbon modelling from soil cores taken at these sites. They report lower soil carbon stocks under *Miscanthus* compared to both arable and grassland control sites although these differences were only found to be significant in the 0–30 cm layer, where arable transition (mean plantation age 6.5 years) suggested a reduction in soil carbon of −0.93 Mg C ha^−1^ yr^−1^ and grassland transition (mean age 7 years) at −3.17 Mg C ha^−1^ yr^−1^; for the 0–100 cm depth, these became 0.05 Mg C ha^−1^ yr^−1^ and −0.69 Mg C ha^−1^ yr^−1^ although differences were not found to be significant when considered over this depth. R. Rowe, A.M. Keith, D. Elias, M. Dondini, P. Smith, J. Oxely and N.P. McNamara (2015, in submission) suggest sampling limitations in many previous studies which typically sample to a fixed depth and do not account for changes in soil bulk density due to land‐use change, and they employed an equivalent soil mass sampling (ESM) strategy in their study, as outlined by Gifford & Roderick (2003). In this technique, sample depth is adjusted to account for soil surface uplift due to belowground Miscanthus biomass. R. Rowe, A.M. Keith, D. Elias, M. Dondini, P. Smith, J. Oxely and N.P. McNamara (2015, in submission) found that apparently larger SOC stocks under *Miscanthus* compared to controls were not seen when using ESM sampling. However, while care was taken to account for soil bulk density changes under Miscanthus, no similar accommodation is made to account for possible erosion losses under annual arable cultivations used as surrogates for baseline.

### Soil carbon turnover

**Figure 4:**
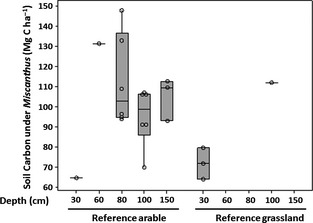
Boxplot of soil organic carbon stocks found under *Miscanthus* results from Table 1. The categories are land use (arable or grassland) and depth of soil that is considered in the SOC content reported in the literature. Varying sample depths reported reflects limited data at greater depths from previous grassland.

**Figure 5:**
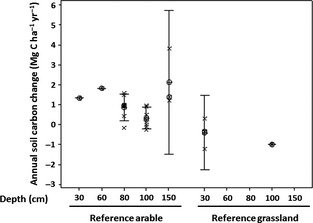
Annual change in soil organic carbon (SOC) under *Miscanthus* from Table 1; as Fig. 3, limited data at greater depths for previous grassland.

**Figure 6:**
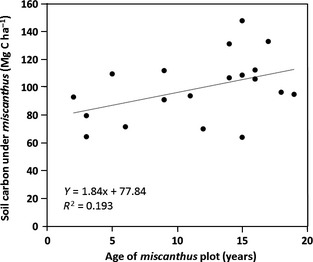
Plantation age vs. SOC stocks under *Miscanthus* from Table 1; slope is 1.84 Mg ha^−1^ yr^−1^ (*R* ^2^ = 0.2).

Even where results suggest maintained or increased SOC, initial disturbance losses after planting *Miscanthus* will still occur. It is important to note, as comprehensively discussed by Agostini *et al*. (2015), that despite SOC changes being generally reported as an annual mean over the age of the plantation, these deltas are unlikely to be constant over time. SOC derived from crop inputs will be lower during the early years of establishment (Zimmermann *et al*., 2012) with disturbance losses of resident C3 carbon outpacing C4 inputs when planted into grassland. Litter drop and root exudates are a function of yield and biomass and will build and reverse this over time although long‐term studies over the potential 15–20 year crop lifetime are notably lacking. Sources of SOC can be investigated using isotopic analysis (Balesdent *et al*., 1987). C4 plants such as *Miscanthus* discriminate less against ^13^C than native C3 plants, and therefore, samples of SOC sequestered under *Miscanthus* will show less depletion of this isotope when compared to an atmospheric standard. Zatta *et al*. (2014) showed that while SOC after conversion of grassland to *Miscanthus* showed no significant difference after 6 years, the isotopic signature showed a clear C4 source demonstrating a rapid turnover in soil carbon with mobilized C3 carbon being quickly replaced, results also shown in other such studies (e.g. Poeplau & Don 2014; Richter *et al*., 2015). Litter input to the soil plays a vital role in sequestering carbon in a mature crop, and overwinter litter drop in *Miscanthus* is reasonably consistent at around 30–35% of aboveground biomass production (Lewandowski *et al*., 2000; Clifton‐Brown *et al*., 2004). The dropped litter accounts for most of the reduction in yield during ripening but is a gain for soil carbon and organic matter and significantly improves overall combustion quality of the harvested biomass. Hansen *et al*. (2004) used stable isotope analysis of soils under two *Miscanthus* plantations to calculate a coefficient of retention for input of carbon from this litter at 26% of the total carbon input for their 9‐year‐old plantation, increasing to 29% for the longer, 16‐year plantation. The data collated in Table 1 suggest a reasonable range of this C4‐C sequestration rate in the top 30 cm to be between 0.5 and 1.5 Mg ha^−1^ yr^−1^ with one outlier at 3.2 Mg ha^−1^ yr^−1^ (Dondini *et al*., 2009a). Combining the, albeit limited, published sample data suggests that stocks of SOC in the top 30 cm on converted grasslands is likely to be higher than converted arable land (Fig. 4), but that the accumulation of SOC occurs faster in conversions from arable soils (Fig. 5). The correlation between plantation age and SOC can be seen in Fig. 6, although the wide scatter (*R* ^2^ = 0.2) likely reflects limited data; the trendline suggests a net accumulation rate of 1.84 Mg C ha^−1^ yr^−1^ with similar levels to grassland at equilibrium.

### Soil carbon spatial modelling across the UK

**Figure 7:**
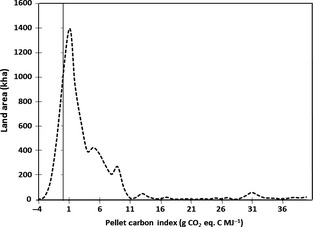
Carbon intensity of *Miscanthus* pellets produced in the UK outside excluded areas described in Lovett *et al*. (2014) and mapped in Fig. 3. Units are g CO_2_‐C equivalent per MJ energy in the furnace. *X*‐axis indicates potential area of land that could produce *Miscanthus* at the carbon intensity index indicated on the *y*‐axis.

Soil carbon stocks are a balance between the soil organic matter decomposition rate and the organic material input each year by vegetation, animal manure, or any other organic input. In *Miscanthus* plots, the difference between peak yield and harvest offtake can be used to calculate the soil carbon input from leaf fall and stubble after harvest and estimates of root turnover (Hansen *et al*., 2004). If the previous land use and soil organic carbon level is known, then the new soil carbon content of land converted to *Miscanthus* plantations can be estimated using models calibrated by field experiments, either in arable land (i.e. Dondini *et al*., 2009a) or in pasture (i.e. Zatta *et al*., 2014); this can be carried out spatially for the entire UK land area and verified by the other published data in Table 1. The cohort model (Bosatta & Ågren, 1985) is used in Fig. 3b to calculate the mean annual SOC change for each km^2^ grid square and its spatial distribution, and the model uses initial soil carbon from the Harmonized World Soil Data Base (HWSD) and the predicted SOC input over 15 years of *Miscanthus* cropping. Figure 7 shows the histogram of SOC change for the UK on land not excluded by Lovett *et al*. (2014) for the first three 15‐year crop cycles of *Miscanthus*. Milner *et al*. (2015) give more details of this and suggest that 99.6% of land within these constraints planted with *Miscanthus* following economic scenarios of Alexander *et al*. (2014) would see net gains in SOC between 1.5 and 2.5 Mg C ha^−1^ yr^−1^, and the slope of Fig. 5 at 1.84 Mg ha^−1^ yr^−1^ fits well within this range. Hillier *et al*. (2009) give a detailed comparison of GHG emissions and SOC changes based on yield predictions for SRC poplar, *Miscanthus*, OSR, and winter wheat with clear overall GHG benefits being seen with *Miscanthus* and SRC on both arable and grassland.

## Chemical requirements

### Fertilizer

Nutrient offtakes at spring harvest in *Miscanthus* are low, and it is therefore generally unfertilized in commercial production except possibly during establishment where initial soil nutrient status is low. Unnecessary use of nitrogen fertilizer reduces the sustainability of biomass production and GWP offset; therefore, understanding where application is necessary and at what specific rates is important. Cadoux *et al*. (2012) reviewed nutrient offtake in mature *Miscanthus* harvests in 27 studies over 10 countries and found a median content of 4.9 g N (kg DM)^−1^ when harvested in the early spring. Given a typical UK offtake of 10–15 Mg DM ha^−1^ yr^−1^, the annual export of organic nitrogen from a site in harvest material would range between 49 and 73.5 kg N ha^−1^. Accounting for an atmospheric N deposition rate of 35–50 kg N ha^−1^ yr^−1^ (Goulding *et al*., 1998) suggests that *Miscanthus* is unlikely to benefit greatly from inputs of N unless it was being established in very low fertility soils; for example, an optimum application of 100 kg N ha^−1^ was seen to give significant yield benefits on a low‐fertility sandy loam soil by Shield *et al*. (2014). Shield *et al*. (2014) emphasized that soil nutrient status prior to establishment was key to determining the need for fertilizer, as are ongoing circumstances. Lewandowski & Schmidt (2006) reported that compared to triticale or reed canary grass, *Miscanthus* showed far higher N‐use efficiency and did not respond well to high concentrations of N fertilizer. Maximum yields were observed with no fertilizer but with existing plant available N in the soil (mineralized) at 50 kg N ha^−1^; higher applications of N fertilization (above 114 kg N ha^−1^ yr^−1^) were detrimental to crop performance, particularly where soil water was in short supply. Lewandowski *et al*. (2000) reviewed 19 *Miscanthus* field trials across Europe and reported that there was little response to N fertilizer after the second or third year, although there was some suggestion that early rhizome development may benefit from a low level of application where soils may be low in available N to begin with. This very low demand for added fertilizer was reported by Christian *et al*. (2006) who used 15N isotope‐enriched nitrogen fertilizer applied at 60 kg N ha^−1^ to study uptake during the establishment phase following planting. Only 23 kg N ha^−1^ of the total 117 kg N ha^−1^ taken up by the developing crop was found to have come from the fertilizer, and 80% had come from mineralization of soil organic matter of the former grassland or atmospheric deposition. There is a growing body of evidence to suggest some level of bacterial nitrogen fixation associated with *Miscanthus* (Davis *et al*., 2010; Dohleman *et al*., 2012). Nitrogenase activity has been found in both rhizomes and surrounding soil bacteria (Eckert *et al*., 2001; Miyamoto *et al*., 2004) with isotope analysis revealing high levels of biologically fixed nitrogen in *Miscanthus* biomass, particularly in the first year of establishment (Keymer & Kent, 2013). Christian *et al*. (2008) followed their *Miscanthus* crop for 14 years under three application regimes, zero, 60, and 120 kg N ha^−1^ yr^−1^, and concluded that there was no yield response from the application of the N fertilizer although monitoring of soil fertility and offtake did suggest, in these soils at least, a benefit from additions of phosphate (7 kg P ha^−1^ yr^−1^) and potassium (100 kg K ha^−1^ yr^−1^). One trade‐off to this low nitrogen requirement is that emissions and leaching may initially rise following planting into highly fertilized land or grassland killed in preparation for conversion (Christian & Riche, 1998; Behnke *et al*., 2012) as *Miscanthus* is unlikely to utilize all the available nutrients in the establishing year. There may, as mentioned in Heaton *et al*. (2010), be a case made for trials of some suitable cover crop to be planted during the transition to take advantage of these resources.

### Pesticide

Despite studies finding some incidence of agricultural disease in *Miscanthus* (Christian *et al*., 1994; O'Neill & Farr, 1996; Ahonsi *et al*., 2010), it does not appear to have become a significant problem after more than a decade of commercial growing in the UK, and pesticides are still not generally considered necessary. Lamptey *et al*. (2003) found that while *Miscanthus* (*M. sinensis* in this case) was susceptible to yield losses from infection with Cereal Yellow Dwarf Virus after being inoculated with them in laboratory experiments, it was more resistant than other energy grasses in their study. All were difficult to infect once the plants had got past the seedling stage but of 18 *Miscanthus* plants none were found to become infected when exposed to the virus after stem extension. Even during its susceptible seedling stage infection was only 33% despite deliberate inoculation, compared to almost 100% for *Phalaris arundinacea* and *Echinochloa crus‐galli*. Lamptey *et al*. (2003) did warn, though, that conventional rhizome propagation and translocation of *Miscanthus* run the risk of disease transfer between sites; care must be taken that crops sourced for rhizomes are disease free as pesticide control on field crops would be uneconomic and undesirable for sustainable biomass production. The results of such infections were seen in a *Miscanthus* field trial in central Italy (Beccari *et al*., 2010) where 90% of the transplanted rhizomes failed to establish due to fungal infection by *Fusarium spp*. and *Mucor hiemalis*. Field contamination and improper rhizome storage (high temperature and humidity) were cited as likely factors and *Miscanthus* litter buried in soil have been found previously to contain *Fusarium* spores (Gams *et al*., 1999). Despite these possible challenges, disease incidence is extremely low with the 14‐year production life with no pesticide application of Christian *et al*. (2008) being typical.

### Herbicide

Once established, *Miscanthus* competes vigorously with weed species, litter build up below the canopy aids suppression, and the fast closing canopy reduces light available to competitors (Lewandowski *et al*., 2000; Christian *et al*., 2008). In the establishing years however, and particularly where grassland is converted, chemical weed control is essential (Jørgensen, 2011). Control is generally accomplished through conventional pre‐ and postemergent herbicides, sometimes combined with timely application of glyphosate immediately prior to new *Miscanthus* shoot emergence, allowing weed species some time to develop before application. Competition from grassland weeds in this type of land‐use transition can be challenging in the early years (Clifton‐Brown *et al*., 2007), and land‐use change follows the conventional practice of glyphosate spraying of the existing vegetation, sometimes in two rounds, before ploughing, soil preparation, and planting. Christian *et al*. (2008) offer rare documentation of their complete herbicide history over a 14‐year *Miscanthus* study, demonstrating that herbicide weed control was not necessary every year with the bulk of their herbicide mixes applied in years one and four with spring application of glyphosate only in years 4 and 13, and they note the effectiveness of the *Miscanthus* canopy structure and litter layer in natural weed suppression.

## Soil nitrous oxide (N_2_O) emission

N_2_O has a global warming potential 298 times greater over 100 years than CO_2_ (IPCC, 2007), and agriculture is the largest producer of this gas (Williams *et al*., 2010; Reay *et al*., 2012). When comparing *Miscanthus* to more usual annual crop rotations, it generally presents lower N_2_O emission although it is well known that N_2_O can be particularly challenging to scale from highly variable individual measurements to landscape sums (Rochette & Eriksen‐Hamel, 2008; Jones *et al*., 2011). Drewer *et al*. (2012) compared both *Miscanthus* and SRC willow to arable rotations of wheat and oil seed rape (OSR), reporting that despite this high variability in the results, the mean N_2_O flux rates were around five times higher under the annual crops than under the unfertilized perennial bioenergy crops with differences being highly significant. They also investigated the effects of adding fertilizer to the *Miscanthus* and OSR control plots at 50 kg N ha^−1^. Before application, *Miscanthus* flux rates were around zero compared to OSR at 300 *μ*g N_2_O‐N m^−2^ h^−1^; emissions began to rise within 24 h of the treatment with the highest flux rate measured after 36 h. *Miscanthus* N_2_O emission rates rose to a maximum of 330 *μ*g N_2_O‐N m^−2^ h^−1^ compared to 2350 *μ*g N_2_O‐N m^−2^ h^−1^ from the OSR. Emissions declined steadily from there, and after 8 days, no significant difference could be found between the two sites. This transient increase in N_2_O following fertilization has been reported in several studies, and Gauder *et al*. (2012) measured emissions rising by a factor of four between fertilized and unfertilized *Miscanthus*, although these were still only around 30% of a fertilized maize comparison. Jørgensen *et al*. (1997), however, measured flux rates from fertilized *Miscanthus* at twice that of winter rye during April and November, though still only around 6% of the gross fossil fuel CO_2_ offset potential they did represent about 1.5% of the mass of N application, exceeding the IPCC tier 1 expectation of 1% (IPCC, 2007) this was corroborated by Behnke *et al*. (2012) who found N_2_O emissions between 1.1 and 2.4% of applied N. Roth *et al*. (2015) calculated the yield response to fertilizer necessary to offset the GWP of this increased N_2_O production. They carried out application trials at 63 and 125 kg N ha^−1^ on a 15‐year‐old crop and concluded that the increased biomass yields they observed did outweigh increased N_2_O emissions; however, it must be considered that their harvest was from a single year and took place in November where biomass could be in the region of 30% greater than the more usual spring harvest. This might suggest that yield gains are found in increased leaf biomass rather than stem which would not translate into harvested biomass for energy or figure in fossil fuel offset as in commercial practice, leaves are ideally lost over winter.

Roth *et al*. (2013) compared newly established and long‐term *Miscanthus* plantations to 18‐year‐old grassland in Ireland. They found that N_2_O flux rates from unfertilized *Miscanthus* are similar to unfertilized, ungrazed *Lolium* grassland although they do suggest higher rates during the early establishment period when planted into previous grassland. They calculated cumulative fluxes under newly established *Miscanthus* at 614 g N_2_O‐N ha^−1^ yr^−1^, less from the established long‐term *Miscanthus* at 378 g N_2_O‐N ha^−1^ yr^−1^ and lowest from the grassland at 217 g N_2_O‐N ha^−1^ yr^−1^. However, being both unfertilized and ungrazed, this grassland is an unrealistic comparison for commercial agriculture. Clayton *et al*. (1997) calculated a figure 10 times higher at 2.94 kg N_2_O‐N ha^−1^ yr^−1^ for fertilized, ungrazed *Lolium* grassland, while Oenema *et al*. (1997), studying intensively managed, grazed grassland, found emissions rising still further, ranging from 10 to 40 kg N_2_O‐N ha^−1^ yr^−1^ as the effects of urine, trampling and dung release around three times as much N_2_O as mown grassland (Velthof *et al*., 1996; Rafique *et al*., 2011).

### Carbon intensity in energy production – life cycle assessment

In theory, burning biomass for energy should be carbon neutral as carbon released to the atmosphere was previously fixed from it during photosynthesis. Greenhouse gas benefits lie in reduced fossil fuel use and associated CO_2_ emission. In the case of crops or forest managed specifically for bioenergy, there are additional energy inputs and associated GHG costs required for the production process that must be considered. Any anthropological intervention in the process of growing vegetation, changes in land cover, and tillage disturbance, using agrochemicals or altered water balances, leads to changes in the soil's physical and chemical properties. Therefore, the cultivation of feedstock for bioenergy will create some GHG emissions which need to be compared to the land use they replace to estimate the net impact on the atmosphere. The embedded carbon in the machinery and plant manufacture, energy use in cultivation, agrochemicals, transport, and processing/conversion of feedstock into fuel also need to be added to the GHG cost of bioenergy in life cycle analyses (LCA).

Hastings *et al*. (2009) compared *Miscanthus* production to fossil fuels using a metric of g CO_2_‐C equivalent emissions per MJ of energy at the furnace. For fossil fuels, they included the cost of exploration, production, processing, and delivery to the furnace and for *Miscanthus* biomass; it was plant propagation to the furnace. Their LCA included the impact on soil carbon per ha of land and used crop yields as reported in Hastings *et al*. (2013) to calculate soil input and energy yield. Establishment costs were spread over a 15‐year crop lifetime and followed the current practice of rhizome propagation with full tillage and two herbicide applications during establishment. It assumed annual cutting, drying in the field in a swath, high‐density bailing and pelleting, and nitrogen fertilizer sufficient to balance the harvest offtake minus N deposition. The IPCC tier 1 N_2_O emission factor of 1% of applied N fertilizer was used with the assumption that production emissions are from European producers. This results in an amortized GHG establishment cost of 124 kg C ha^−1^ y^−1^ and a yield‐related annual GHG cost of 57 kg C Mg^−1^ of crop. The results in Fig. 3c show most of the land in the UK could produce *Miscanthus* biomass with a carbon index that is substantially lower, at 1.12 g CO_2_‐C equivalent per MJ energy in the furnace, than coal (33), oil (22), LNG (21), Russian gas (20), and North Sea gas (16) (Bond *et al*., 2014), thus offering large potential GHG savings over comparable fuels even after accounting for variations in their specific energy contents. Felten *et al*. (2013) found *Miscanthus* energy production (from propagation to final conversion) to offer far higher potential GHG savings per unit land area when compared to other bioenergy systems. They found *Miscanthus* (chips for domestic heating) saved 22.3 ± 0.13 Mg CO_2_‐eq ha^−1^ yr^−1^ compared to rapeseed (biodiesel) at 3.2 ± 0.38 and maize (biomass, electricity, and thermal) at 6.3 ± 0.56. Only the low‐input *Miscanthus* was found to be effectively a CO_2_ sink. Styles & Jones (2007) calculated GHG savings for Miscanthus in Ireland at 35 Mg CO_2_‐eq ha^−1^ yr^−1^, while Brandao *et al*. (2011) gave a figure of 11.01 for the UK. Of course these savings are determined by the specific energy source they offset and comparisons do not always account for displaced production. Styles *et al*. (2015) investigated the effects of indirect land‐use change, that is considering GHG emissions from the production of food displaced by bioenergy feedstock production. They found only *Miscanthus* and rotational maize offered GHG savings when these indirect land‐use change (iLUC) impacts were considered and the percentage of displaced production that was directly replaced determined a threshold. Typically replacing 2–14% for food crops or grassland diverted into anaerobic digestion negated potential GHG savings, whereas it was around 85% for pelletized *Miscanthus*. The GHG benefits for rotational maize were, however, heavily offset by ecosystem service impacts due to intensive production, and of the six bioenergy crop systems investigated, *Miscanthus* was shown to offer the greatest benefits in ecosystem service provision. It was stressed, though, that these positive effects could be localized, consideration needed to be given where production might be displaced to and the impacts of any land‐use changes incurred. The importance of understanding indirect land‐use change was also highlighted by Tonini *et al*. (2012) who used sensitivity analysis to show that uncertainties around this were significant determinants in LCA results. They compared four conversion pathways (AD, gasification, small‐scale CHP, and large‐scale cofiring with coal) for ryegrass, willow, and *Miscanthus* and found that when considering their Danish systems, only large‐scale cofiring of *Miscanthus* and willow offered real GHG savings compared to fossil fuel alternatives.

## Water balance

### Water‐use efficiency

*Miscanthus* has higher water‐use efficiency (WUE) compared to more conventional C3 crop species, and even some other C4 crops which typically produce more biomass per unit of water transpired (Long, 1983). Beale *et al*. (1999) investigated WUE in field trials of *Miscanthus* and another potential C4 biomass crop, *Spartina cynosuroides,* under both rain‐fed and irrigated conditions; they estimated the ratio of aboveground biomass to water use for *Miscanthus* under rain‐fed conditions at 9.2 g DM (kg H_2_O)^−1^ compared to 6.8 g DM (kg H_2_O)^−1^ for *S. cynosuroides*. Both crops appeared to become less efficient under irrigation, down by 15% for *Miscanthus* to 7.8 g DM (kg H_2_O)^−1^ and 25% for *S. cynosuroides* to 5.1 g DM (kg H_2_O)^−1^, possibly reflecting greater canopy evaporation under the irrigation regime. Beale *et al*. (1999) compared their results to the water‐use efficiency of a C3 biomass crop, *Salix viminalis,* reported in Lindroth *et al*. (1994) and Lindroth & Cienciala (1996), and suggest that WUE for *Miscanthus* could be around twice that of this willow species. Clifton‐Brown & Lewondowski (2000) reported figures from 11.5 to 14.2 g total (above‐ and belowground) DM (kg H_2_O)^−1^ for various *Miscanthus* genotypes in pot trials, and this compares to figures calculated by Ehdaie & Waines (1993) with seven wheat cultivars who found WUE between 2.67 and 3.95 g total DM (kg H_2_O)^−1.^ Converting these *Miscanthus* values to dry matter biomass per hectare of cropland would see ratios of biomass to water use in the range of to 78–92 kg DM ha^−1^ (mm H_2_O)^−1^. Richter *et al*. (2008) modelled harvestable yield potentials for *Miscanthus* from 14 UK field trials and found soil water available to plants was the most significant factor in yield prediction, and they calculated a DM yield to soil available water ratio at 55 kg DM ha^−1^ (mm H_2_O)^−1^, while just 13 kg DM ha^−1^ was produced for each 1 mm of incoming precipitation, likely related to the high level of canopy interception and evaporation. Even by C4 standards these efficiencies are high, as seen in comparisons to field measurements averaging 27.5 ± 0.4 kg aboveground DM ha^−1^ (mm H_2_O)^−1^ for maize (Tolk *et al*., 1998).

### Soil water balance

However, despite impressive efficiency figures, accumulating biomass at the rapid rate that makes *Miscanthus* interesting as an energy crop will inevitably lead to increased demand for water and consideration needs to be given to water availability when locating plantations (Vanloocke *et al*., 2010). When Yaeger *et al*. (2013) compared *Miscanthus* to corn and soya bean grown in the American mid‐West, they found that *Miscanthus* had effectively a 2‐month longer season of transpiration which meant a reduction in soil water reserves during low rainfall. This reduction can be exacerbated by the dense canopy of *Miscanthus* which intercepts more rain allowing more evaporation at the leaf level and less throughfall to the soil compared to some other crops. Stephens *et al*. (2001) modelled reductions in hydrologically effective rainfall (HER), that is rainfall that becomes incorporated into the soil under *Miscanthus* and willow compared to permanent grass and winter wheat. Results showed reductions in HER under *Miscanthus* were lower than those for willow SRC but still large at between 100 and 120 mm yr^−1^; using their estimate for 0.10 Mha of energy crops reducing HER by 150 mm (average across both crops was 140–180 mm yr^−1^) meant 0.35 Mha would reduce rainfall reaching the soil by 0.7% of total UK rainfall. Blanco‐Canqui (2010) point out that this water‐use and nutrient efficiency can be a boon on compacted, poorly drained acid soils, highlighting their possible suitability for marginal agricultural land. The greater porosity and lower bulk density of soils under perennial energy grasses, resulting from more fibrous, extensive rooting systems, and reduced ground disturbance, improves soil hydraulic properties, infiltration, hydraulic conductivity, and water storage compared to annual row crops. There may be potentially large impacts on soil water where plantation size is mismatched to water catchment or irrigation availability but note that increased ET and improved ground water storage through increased porosity could be beneficial during high rainfall with storage capability potentially increased by 100 to 150 mm. There is also a benefit of reduced chemical inputs and nitrate leaching associated with *Miscanthus,* significantly improving water quality running off farmland (Christian & Riche, 1998; Curley *et al*., 2009). McIsaac *et al*. (2010) reported that inorganic N leaching was significantly lower under unfertilized *Miscanthus* (1.5–6.6 kg N ha^−1^ yr^−1^) than a maize/soya bean rotation (34.2–45.9 kg N ha^−1^ yr^−1^). They also reported that soils under *Miscanthus* were drier and calculated increased evapotranspiration from the *Miscanthus* at 104 mm yr^−1^. Finch *et al*. (2004) studied UK energy grasses and willow SRC and compared them to existing grassland and arable. They found that in years of sufficient rainfall, *Miscanthus* is likely to use less or the same water as existing agricultural land. In drought years, although *Miscanthus* was likely to impact more on soil water deficits due to increased interception and rooting depth, this could lead to reduced groundwater recharge rates in drier years or reduced winter run‐off in wetter conditions.

## Biodiversity

As *Miscanthus* is an agricultural crop, its place in an agricultural landscape of fields, margins, and farm woodland should be considered in terms of its potential to increase or decrease resources for wildlife in land‐use transitions. How does it compare to existing land use or other potential energy crops? Felten & Emmerling (2011) compared earthworm abundance under a 15‐year‐old *Miscanthus* plantation in Germany to cereals, maize, OSR, grassland, and a 20‐year‐old fallow site (after previous cereals). Species diversity was higher in *Miscanthus* than that in annual crops, more in line with grassland or long‐term fallow with management intensity seen to be the most significant factor; the lower ground disturbance allowed earthworms from different ecological categories to develop a more heterogeneous soil structure. The highest number of species was found in the grassland sites (6.8) followed by fallow (6.4), *Miscanthus* (5.1), OSR (4.0), cereals (3.7), and maize (3.0) with total individual earthworm abundance ranging from 62 m^−2^ in maize sites to 355 m^−2^ in fallow with *Miscanthus* taking a medium position (132 m^−2^), although differences in abundance were not found to be significant between land uses. There is some trade‐off in this advantage for the earthworms however; the high‐nitrogen‐use efficiency and nutrient cycling which reduces the need for nitrogen fertilizer and its associated environmental harm means that, despite large volumes being available, *Miscanthus* leaf litter does not provide a particularly useful food resource due to its low‐nitrogen, high‐carbon nature (Ernst *et al*., 2009; Heaton *et al*., 2009) and earthworms feeding on this kind of low‐nitrogen material have been found in other studies to lose overall mass (Abbott & Parker, 1981). In contrast, though, the extensive litter cover at ground level under *Miscanthus* compared to the bare soil under annual cereals was suggested to be a potentially significant advantage for earthworms in soil surface moisture retention and protection from predation.

Semere & Slater (2007a,b) sampled an exhaustive range of aboveground indicator species at five sites in Herefordshire, UK. They compared results between *Miscanthus*, reed canary grass, and switchgrass and found *Miscanthus* to contain high levels of diversity in comparison with the other energy grasses; particularly evident in terms of beetles, flies, and birds, with breeding skylarks and lapwings being recorded in the crop itself. It was pointed out by the authors, however, that although the overwinter vegetative structure provided an important cover and habitat resource, it was the noncrop weed species in and around the field sites that underpinned the food webs supporting the bird species. This link between crop density, weed content, and food resources for birds was again demonstrated by Dauber *et al*. (2015) who recorded the abundances of ground fauna, beetles, spiders, etc., at 14 mature *Miscanthus* sites in SE Ireland. They found light penetration through the canopy directly related to within crop biodiversity, with *Miscanthus* planted on previous grassland showing higher levels of biodiversity compared to that planted on former arable.

This trade‐off between crop success and within‐crop biodiversity is to be expected. For an economic return, the most efficient capture of light by the crop canopy will inevitably reduce noncrop weed resources for other species. However, *Miscanthus* offers environmental benefits in structural heterogeneity, low chemical inputs, and overwinter ripening providing near continuous cover. Particularly in a landscape of high‐input arable production, *Miscanthus* has the potential to offer a 10‐ to 15‐year break crop allowing the soil and surrounding field margins time to recover from intensive production. Bellamy *et al*. (2009) looked at bird species and their food resources at six paired sites in Cambridgeshire comparing *Miscanthus* plantations up to 5 years old with winter wheat rotations in both the winter and summer breeding seasons. The authors found that *Miscanthus* offered a different ecological niche during each season; most of the frequently occurring species in the winter were woodland birds, whereas no woodland birds were found in the wheat; in summer, however, farmland birds were more numerous. More than half the species occurring across the sites were more numerous in the *Miscanthus*, 24 species recorded compared to 11 for wheat. During the breeding season, there was once again double the number of species found at the *Miscanthus* sites with individual abundances being higher for all species except skylark. Considering only birds whose breeding territories were either wholly or partially within crop boundaries, a total of seven species were found in the *Miscanthus* compared to five in the wheat with greater density of breeding pairs (1.8 vs. 0.59 species ha^−1^) and also breeding species (0.92 vs. 0.28 species ha^−1^). Two species were at statistically significant higher densities in the *Miscanthus* compared to wheat, and none were found at higher densities in the wheat compared to *Miscanthus*. As discussed, the structural heterogeneity, both spatially and temporally, plays an important role in determining within‐crop biodiversity, autumn‐sown winter wheat offers little overwinter shelter with ground cover averaging 0.08 m tall and very few noncrop plants, whereas the *Miscanthus,* at around 2 m, offered far more. In the breeding season, this difference between the crops remained evident; the wheat fields provided a uniform, dense crop cover throughout the breeding season with only tram lines producing breaks, whereas the *Miscanthus* had a low open structure early in the season rapidly increasing in height and density as the season progressed. Numbers of birds declined as the crop grew with two bird species in particular showing close (though opposite) correlation between abundance and crop height; red‐legged partridge declined as the crop grew, whereas reed warblers increased, and these warblers were not found in the crop until it had passed 1 m in height, even though they were present in neighbouring OSR fields and vegetated ditches. In conclusion, the authors point out that, for all species combined, bird densities in *Miscanthus* were similar to those found in other studies looking at SRC willow and set‐aside fields, all sites had greater bird densities than conventional arable crops.

It is through these added resources to an intensive agricultural landscape and reductions in chemical and mechanical pressure on field margins that *Miscanthus* can play an important role in supporting biodiversity but must be considered complementary to existing systems and the wildlife that has adapted to it. Clapham *et al*. (2008) reports, as do the other studies here, that in an agricultural landscape, it is in the field margins and interspersed woodland that the majority of the wildlife and their food resources are to be found, and the important role that *Miscanthus* can play in this landscape is the cessation of chemical leaching into these key habitats, the removal of annual ground disturbance and soil erosion, improved water quality, and the provision of heterogeneous structure and overwinter cover.

## Summary statements

Based on the literature evidence reviewed above, we present here a number of summary statements addressing concerns and questions around the environmental sustainability of *Miscanthus* production in the UK.

### Potential UK land availability

- By planting in appropriate locations, government targets of 0.35 Mha of dedicated energy crops could be sustainably met by *Miscanthus* production without impacting essential food production.
- 0.35 Mha (2007 Biomass Strategy) would provide the energy equivalent to 1.67 Mt of oil each year (1.17% of total energy).
- 0.72–2.8 Mha (2012 Bioenergy Strategy) would provide the equivalent of 3.44–13.38 Mt of oil yr^−1^ (2.41–9.39% of total energy).

### Soil carbon

- Former arable land converted to *Miscanthus* is most likely to lead to no change or an accumulation of soil organic carbon (SOC), becoming comparable to an agricultural grassland within the lifetime of the crop. *Miscanthus* contributes 0.98 ± 0.14 Mg C4‐C ha^−1^ yr^−1^ through litter drop and root turnover.
- Converting semi‐permanent grassland to *Miscanthus* by traditional establishment (spraying, ploughing, tilling, and planting) results in an initial short‐term soil carbon loss which is recovered as the crop matures.

### Chemical inputs

- Nitrogen fertilizer is unnecessary and can be detrimental to sustainability, unless planted into low‐fertility soils where early establishment will benefit from additions of around 50 kg N ha^−1^.
- Early season herbicide application for weed control is essential in the establishing years but becomes redundant as the crop matures, other pesticides are not needed.

### GHG cost of energy production

- When considering the entire energy supply chain burning, *Miscanthus* produces far less GHG per MJ of energy than fossil fuels; 1.12 g CO_2_‐C eq. compared to coal (33), oil (22), gas (16–22).

### Nitrous oxide (N_2_O) emission

- N_2_O emissions can be five times lower under unfertilized *Miscanthus* than annual crops, and up to 100 times lower than intensive pasture land.
- Inappropriate nitrogen fertilizer additions can result in significant increases in N_2_O emission from *Miscanthus* plantations, exceeding IPCC emission factors although these are still offset by potential fossil fuel replacement.

### Water balance

- Water‐use efficiency is among the highest of any crop, in the range of 7.8–9.2 g DM (kg H_2_O)^−1^.
- Overall, water demand will increase due to high biomass productivity and increased evapotranspiration at the canopy level (e.g. ET up from wheat by 100–120 mm yr^−1^).
- Improved soil structures mean greater water‐holding capacity (e.g. up by 100–150 mm), although soils may still be drier in drought years.
- Reduced run‐off in wetter years, aiding flood mitigation and reducing soil erosion.
- Drainage water quality is improved, and nitrate leaching is significantly lower than arable (e.g. 1.5–6.6 kg N ha^−1^ yr^−1^ *Miscanthus*, 34.2–45.9 maize/soya bean).

### Biodiversity

- *Miscanthus* adds structural resources to agricultural landscapes, provides overwinter cover, and increases temporal variability which is accessed by different bird species in different seasons.
- Earthworm diversity and abundance is improved in arable soils and comparable to grassland soils although biomass may be reduced through poorer food quality.
- Reduced chemical inputs improve headland and field boundary quality for wildlife.
- Unpalatability of leaf litter and harvest residue removes the need for pest control but trade‐off food resources for invertebrates within the cropped area are only provided by interspersed weed species are limited to weed species.

## Conclusion

This study distils a large body of literature into simple statements around the environmental costs and benefits of producing *Miscanthus* in the UK, and while there is scope for further research, particularly around hydrology at a commercial scale, biodiversity in older plantations or higher frequency sampling for N_2_O in land‐use transitions to and from *Miscanthus*, clear indications of environmental sustainability do emerge. Any agricultural production is primarily based on human demand, and there will always be a trade‐off between nature and humanity or one benefit and another; however, the literature suggests that *Miscanthus* can provide a range of benefits while minimizing environmental harm. Consideration must be given to appropriateness of plantation size and location, whether there will be enough water to sustain its production and the environmental cost of transportation to end‐users; its role as a long‐term perennial crop in a landscape of rotational agriculture must be understood so as not to interfere with essential food production. There is nothing new in these considerations, they lie at the heart of any agricultural policy, and decision‐makers are familiar with these issues; the environmental evidence gathered here will help provide the scientific basis to underpin future agricultural policy. It is only through an understanding at the government level that uptake of *Miscanthus* will be able to fulfil its potential in the UK bioenergy sector. Despite clear environmental benefits and developing supply chains, uptake of *Miscanthus* production remains low among UK farmers and there is much inertia to overcome. Financial considerations are paramount in farmers’ willingness to adopt novel crops and production practices and uncertainty around grant funding, establishment costs, potential yields, and sale price limits confidence (Sherrington *et al*., 2008; Adams *et al.,* 2011). There is, however, growing awareness of the bigger picture of environmental stewardship and climate change mitigation (Glithero *et al*., 2013) and farmers stress the need for clear, unbiased information on all aspects of bioenergy; from the entire cycle of crop management and marketing to end use, biomass boilers for on‐farm use and local energy supply. There is a problem of ‘chicken and egg’ in developing these markets; farmers need the incentive of a mature market to sell into to encourage their uptake of these crops, whereas energy producers need a large‐scale, secure, predictable supply of biomass to invest in the technologies to utilize them. Without top‐down intervention, and policy stability, it will be difficult for a ‘critical mass’ of growers to develop to provide confidence in energy crop supply.
